# Strengthening the Brain—Is Resistance Training with Blood Flow Restriction an Effective Strategy for Cognitive Improvement?

**DOI:** 10.3390/jcm7100337

**Published:** 2018-10-09

**Authors:** Alexander Törpel, Fabian Herold, Dennis Hamacher, Notger G. Müller, Lutz Schega

**Affiliations:** 1 Institute III, Department of Sport Science, Otto von Guericke University Magdeburg, Zschokkestr. 32, 39104 Magdeburg, Germany; dennis.hamacher@ovgu.de (D.H.); lutz.schega@ovgu.de (L.S.); 2 Research Group Neuroprotection, German Center for Neurodegenerative Diseases (DZNE), Leipziger Str. 44, 39120 Magdeburg, Germany; Fabian.Herold@dzne.de (F.H.); notger.mueller@dzne.de (N.G.M.); 3 Center for Behavioral Brain Sciences (CBBS), Universitätsplatz 2, 39106 Magdeburg, Germany; 4 Department of Neurology, Medical Faculty, Otto von Guericke University, Leipziger Str. 44, 39120 Magdeburg, Germany

**Keywords:** cognition, strength training, blood flow restriction, neuroplasticity

## Abstract

Aging is accompanied by a decrease in physical capabilities (e.g., strength loss) and cognitive decline. The observed bidirectional relationship between physical activity and brain health suggests that physical activities could be beneficial to maintain and improve brain functioning (e.g., cognitive performance). However, the exercise type (e.g., resistance training, endurance training) and their exercise variables (e.g., load, duration, frequency) for an effective physical activity that optimally enhance cognitive performance are still unknown. There is growing evidence that resistance training induces substantial brain changes which contribute to improved cognitive functions. A relative new method in the field of resistance training is blood flow restriction training (BFR). While resistance training with BFR is widely studied in the context of muscular performance, this training strategy also induces an activation of signaling pathways associated with neuroplasticity and cognitive functions. Based on this, it seems reasonable to hypothesize that resistance training with BFR is a promising new strategy to boost the effectiveness of resistance training interventions regarding cognitive performance. To support our hypothesis, we provide rationales of possible adaptation processes induced by resistance training with BFR. Furthermore, we outline recommendations for future studies planning to investigate the effects of resistance training with BFR on cognition.

## 1. Introduction

From the third decade of life, degenerative changes of the human organism increase which leads on the one hand to a reduced physical performance and on the other hand to a decline of cognitive functions. In terms of physical performance, especially the loss of muscle mass [1,2,3,4] contributes to a decrease in muscular strength which, in turn, impairs activities of daily living (e.g., walking) [5,6]. However, musculature is the main effector organ for developing muscular strength which is important to ensure motion respectively locomotion (e.g., walking safely) [7,8,9]. Therefore, the integrity of the musculature and the muscle strength is of great importance throughout the entire life span. Moreover, the mentioned age-related decreases in muscle mass and strength (because of aging) are also associated with morphological losses in the brain and decreased cognitive functions [10,11,12,13]. Because of those changes, especially cognitive functions such as memory and processing speed are negatively affected [14,15,16,17,18]. Furthermore, aging-related changes of the brain are considered risk factors for the development of neurological diseases (e.g., dementia) [19,20]. Dementia is associated with cognitive impairments negatively affecting quality of life and independent living [19,21]. Based on the limited ability of individuals with neurological diseases (e.g., dementia) to live independently, an intensive medical care is needed which, in turn, consumes a large amount of resources of the welfare systems of industrialized nations [22,23,24,25].

So far, no pharmacological interventions are sufficient to treat the mentioned age-associated declines [26,27,28,29,30]. But, there is growing evidence with respect to positive effects of physical activity preventing and treating morphological and functional losses in muscles [31] and the brain [32,33]. In recent years, evidence has emerged emphasizing the existence of a bidirectional relationship between physical performance and brain health [34,35]. For instance, as mentioned above, a decrease in muscular performance is associated with a decrease in cognitive functioning [36,37,38]. Consequently, the bidirectional relationship suggests that physical training (means a structured, planned, dosed, and systematic form of physical activity with the focused aim to increase physical performance and/or health; e.g., through resistance training) may be a valuable intervention strategy to deaccelerate not only physical but also cognitive decline in old age. However, the exercise type (e.g., resistance training, endurance training) and exercise variables (e.g., load, duration, frequency), which would be optimal to efficiently enhance cognitive performance are largely unknown [39,40,41,42,43,44,45,46,47,48,49].

A promising and cost-effective physical intervention strategy [50,51,52] which preserves and enhances both, physical performance (especially with regard to the musculature) [53,54,55,56,57,58,59,60] and cognitive functions [61,62,63,64,65], is resistance training (also known as strength training). The underlying neurobiological mechanisms and effects of resistance training on cognition are described in the following section.

## 2. Effects and Mechanisms of Resistance Training on Cognition

The underlying neurobiological processes which are triggered by resistance exercises and have been related to cognitive performance improvements, are not fully understood, yet [61,65,66]. Based on the promising framework of Stillman et al. [67] about mediators of physical activity (in this case resistance exercises) influencing cognitive performance on different levels (cellular and molecular level, structural and functional level and behavioral/socioemotional level) [67], the current knowledge of possible neurobiological mechanisms contributing to the improvement of cognitive functions in response to resistance training are summarized in the following.

On the cellular and molecular level, a possible key mechanism of resistance training that contributes to cognitive improvements is the pronounced release of the multifaceted acting insulin-like growth factor 1 (IGF-1) [61,62,66,68,69,70]. In response to resistance training, IGF-1 is mainly expressed by the liver (global output, ~70% of total circulating IGF-1), musculature (local output) and the brain (local output) itself [71,72]. Circulating IGF-1 can cross the blood-brain barrier (BBB) which is therefore also available to the brain [71,72]. While an increased IGF-1 level is associated with proliferation, differentiation, survival, and migration of neuronal progenitors [73,74], synaptic processes (e.g., Long-Term Potentiation) [74,75], angiogenesis in the brain, neuroprotection, axon outgrowth, dendritic maturation, and synaptogenesis [72,76], a deficiency of IGF-1 is associated with the risk of harmful cerebrovascular events (e.g., ischemic stroke or impaired neurovascular coupling) [77,78]. Consequently, it is not surprising that a relationship between cognitive functions and IGF-1 level in older individuals [79] and in individuals with mild cognitive impairments was observed [80]. Furthermore, it is assumed that there is a potential relationship between diminished IGF-1 levels and neurodegenerative diseases [73,80,81], which suggests that influencing IGF-1 levels is a promising target for efficient treatments.

In fact, serum IGF-1 concentration levels are increased after a single bout of resistance activities (short-term) [82] and long-term (also known as “chronic”; >2 exercise sessions) resistance training in humans [83,84]. However, currently there is only low evidence postulating a solid relationship between physical exercise-induced modulation of IGF-1 release and cognitive functions [85]. Nevertheless, one study reveals that basal changes of IGF-1 concentrations after a long-term resistance exercise intervention are associated with cognitive performance improvements [83]. Hence, further studies are needed to get a deeper understanding of the relationship of exercise-induced modulation of IGF-1 release and cognition [85].

On the structural level, Fontes et al. [86] observed that in older individuals, the grey matter density increases in the posterior and anterior lobe of the cerebellum, superior frontal gyrus in the frontal lobe and anterior cingulate cortex in the limbic lobe in response to a 12 weeks resistance training [86]. After a 6 months resistance exercise training program, an increase in cortical thickness in posterior cingulate cortex was observed which correlated with improvements in an overall cognition score [87]. Furthermore, in the study of Liu-Ambrose et al. [88], a reduced whole-brain volume after the end of 12 months resistance intervention as compared to control groups (balance and tone group) was noticed [88]. The reduced brain volume might be the consequence of dissolve degenerative changes of the brain such as amyloid plaques [46,88,89]. However, the distinct neuronal adaptions in response to resistance exercise interventions with different exercise variables suggest that a certain dose-response relationship between physical exercise variables and neural adaptations exists, although this dose-response relationship is currently poorly understood and has to be investigated in further studies [42,64,90,91,92,93].

In addition, long-term resistance training is associated with decreased white matter atrophy at follow-up measurements [94] and lower white matter lesions volume was observed after 52 weeks of a resistance training exercise regime [95]. White matter changes are known to influence cognitive performance especially in processing-speed-dependent cognitive tasks [96,97,98,99].

On the functional level, changes can be quantified either by measuring the activity of brain regions (for instance with electroencephalography [EEG], functional near-infrared spectroscopy [fNIRS], or functional magnetic resonance imaging [fMRI]) and/or by testing cognitive functions. Both, brain activity and cognitive functions were investigated after short-term and long-term resistance training to identify beneficial effects of this type of exercise on brain as well as cognitive performance [64]. In response to an acute bout of moderate-load [100] and high-load resistance training, an improvement in cognitive functions (higher number of solved items and lower reaction times in neutral Stroop task condition compared to non-exercising control group) and a decrease in the tissue oxygenation index in left and right prefrontal cortex was observed [101]. In the same manner, it has been shown that resistance training lasting several months can lead to a substantial increase in cognitive functions [62,63,64,83,88,94,102,103]. Furthermore, after a long-term resistance training intervention, a decreased cortical activation in prefrontal areas (lower concentration of oxygenated hemoglobin and total hemoglobin index values measured by fNIRS) during a standardized cognitive test (e.g., Stroop-test) was noticed [104]. A decreased activation in prefrontal areas and a simultaneous increase in cognitive functions may point towards a higher automatization in behavioral tasks or the redistribution of resources in other task-relevant cortical areas. The notion that higher levels of strengths are beneficial for cognitive performance is further supported by numerous cross-sectional studies observing that an improvement of hand grip strength [38,105,106], quadriceps strength [37], leg power [107], or whole body muscle strength [36] are linked to higher cognitive performance. Regarding the longitudinal and cross-sectional studies, the question arises whether (baseline) strength level per se [108] or adaptation processes evoked by regularly conducted resistance training (see above mentioned adaptations on cellular, molecular and structural level) are more beneficial for cognitive performance. Based on the current available scientific literature, we cannot unequivocally answer this question. As shown, there is evidence for both approaches (baseline strength vs. adaptation processes evoked by regularly conducted resistance training). But maybe just the combination of both has positive effects on cognitive functions.

On the behavioral/socioemotional level, the improvements in cognitive functions (e.g., executive functions) and the reduced activity of the prefrontal cortex are, for instance, linked to the functioning of the motor control of activities of daily living such as walking safely [109,110,111,112,113]. This phenomenon underpins the need to persevere the capacity of executive functions especially in older individuals in order to ensure mobility and independent living. Furthermore, because of the relationship between cognitive functions and quality of life [114], improvements in cognitive functions might be associated with an enhanced socioemotional status (e.g., decreased symptoms of depression and anxiety, increased joyful activities of daily living). Here, positive effects of resistance training on quality of life have been noticed [115].

However, concerning the effectiveness of the type of exercise, it was reported that resistance training is less effective than aerobic exercises regarding the improvement of cognitive performance on behavioral/socioemotional level [116] or on functional level regarding the task-related oxygenation of brain regions [101,104]. Nevertheless, there are several strategies to increase the effectiveness of resistance exercise regimes. A potential strategy which is likely to be beneficial to increase the efficiency of resistance training is the application of devices (e.g., cuffs) modulating the blood flow to and away from the muscles. This type of training is known as blood flow restriction training (BFR). So far, the higher effectiveness of resistance training with BFR compared to resistance training without BFR has only been investigated in the context of muscle physiological adaptions and strength improvements [117,118,119]. Whether resistance training with BFR provides also positive neurocognitive effects that are potentially greater than those effects observed after “traditional” resistance training interventions (resistance training without BFR) will be discussed in detail in the following section.

## 3. Resistance Training with Blood Flow Restriction—An Added Value for Cognition?

**Figure 1:**
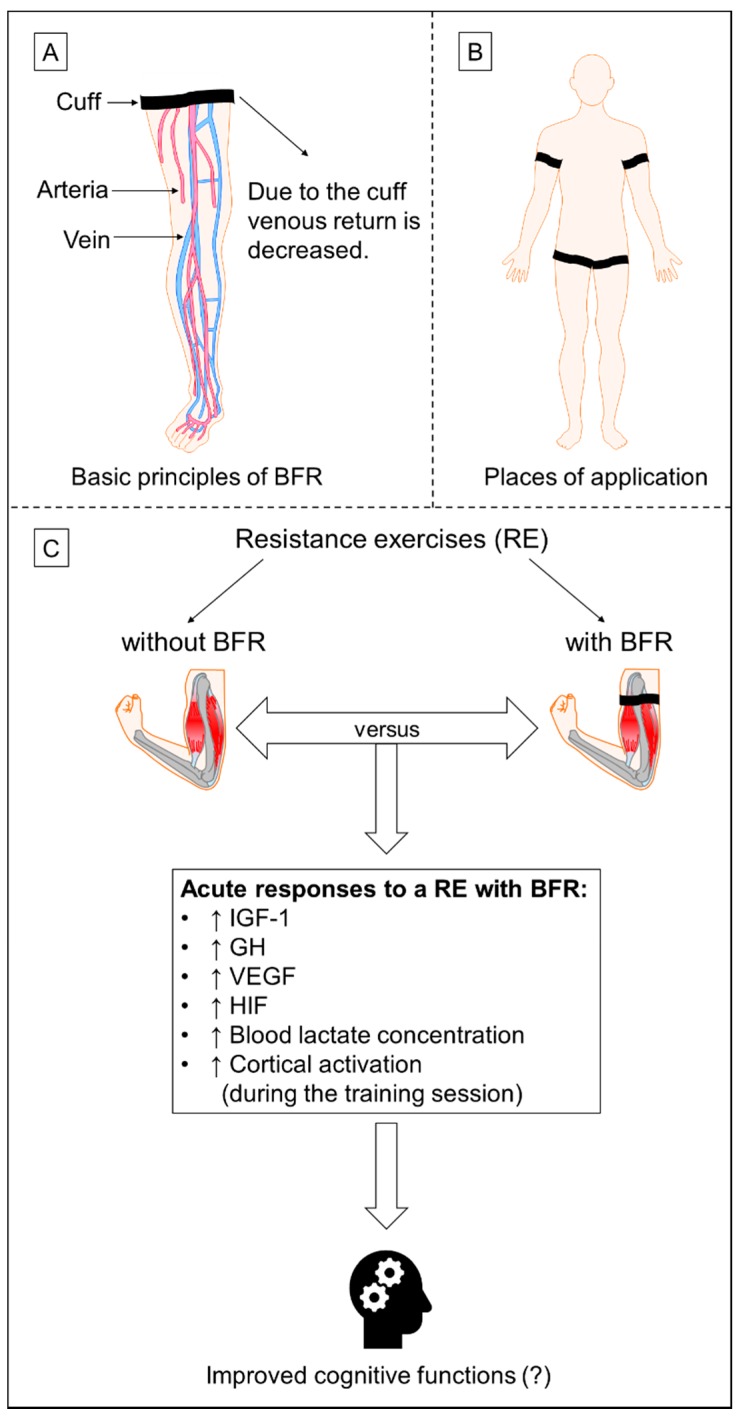
Schematic illustration of (**A**) the basic principles of blood flow restriction, (**B**) the application places of the cuffs for blood flow restriction and (**C**) the possible neurobiological mechanisms of resistance training with blood flow restriction that are likely to contribute to improved cognitive functions; blood flow restriction (BFR), growth hormone (GH), hypoxia-inducible factor (HIF), insulin-like growth factor 1 (IGF-1), resistance training (RE), vascular endothelial growth factor (VEGF).

A way to increase the efficiency of resistance training is the specific manipulation of different exercise variables such as load, volume (repetitions, sets), rest periods, repetition velocity, choice of exercise, order of exercise, frequency or muscle action. [120]. Here, a certain dose-response relationship regarding certain exercise variables (e.g., load) can be observed [61,121,122]. Another, newer “manipulation strategy” to increase the efficiency of resistance training includes the application of hypoxic stimuli [123,124,125,126]. Hypoxic stimulation during resistance exercises could be achieved by applying (i) localized hypoxia or (ii) systemic hypoxia [125]. Localized hypoxia can be achieved with applying BFR which is in the literature also referred to as *occlusion training*. The training method BFR is characterized by the restriction / manipulation of the blood flow to and away from the limbs due to the application of elastic straps or inflatable pressure cuffs (e.g., blood pressure cuffs) to the proximal portion of the limbs (see Figure 1A,B) [117,125,127,128,129,130]. The manipulation of the blood flow especially decreases the venous return, which increases the accumulation of metabolites in the muscle triggering pronounced adaptational processes [117,125,127,128,129,130]. A particular type of BFR is KAATSU where special inflatable cuffs with pressure sensors are used [131]. Even though KAATSU is considered a type of BFR, this term is in a strict sense only applicable if KAATSU-equipment in BFR training is used. As consequence of the special construction of KAATSU-cuffs and their distinct application protocol, it is likely that differences between KAATSU and other BFR methods regarding the physiological stimuli occur. So far, these possible physiological differences between KAATSU and other BFR methods have not been directly and systematically compared. In this manuscript, the term BFR will therefore also include KAATSU training studies.

In general, systemic hypoxia is provided by breathing oxygen-reduced air [125]. Here, the oxygen-reduced air can be applied, for instance, with mask-system hypoxicators or via a stay in special rooms where the fraction of inspired oxygen is decreased (also known as normobaric altitude chambers) [132].

Both, localized hypoxia (induced by BFR) and systemic hypoxia are harmless (when conducted appropriately) and well feasible [133,134,135]. However, due to the cuffs on the limbs during the BFR, petechial haemorrhage beneath the skin and/or numbness of extremities can appear in few cases [125,134,136]. Compared to localized hypoxia (e.g., BFR), systemic hypoxia has the advantage that it is not limited to the limbs [125]. Remarkably, cross-transfer effects in muscles that were not directly affected by the application of blood flow manipulation cuffs were observed in response to resistance exercises with BFR. Both, muscles proximal to the restricted extremities and muscles distal to the restricted extremities experience beneficial effects [137,138]. Systemic-endocrinological (e.g., expression of growth factors) as well as neuronal adaptations (e.g., higher recruitment of supportive muscles because of the increased fatigued muscles under BFR) are discussed for this phenomenon. However, regarding brain adaptions, systemic hypoxia leads to an oxygen deficit directly in the brain which is to a certain extent the decisive stimulus triggering positive neurophysiological adaptations [135,139,140]. In this regard, first studies have shown improved cognitive functions following interventions with systemic, normobaric hypoxia [141,142].

Also, for resistance training with BFR, a first investigation by Sardeli et al. [143] had observed positive effects on cognitive functions (Stroop-test) immediately after a low-load resistance training with BFR (30% of 1RM) [143]. Except for this first investigation of Sardeli et al. [143], there are to our knowledge currently no further studies available (neither short-term nor long-term study) which directly examine the effects of localized hypoxic exposure on cognitive performance. Based on the first hint that localized hypoxia is beneficial for cognition, we want to outline several reasons why localized hypoxia during a resistance training (e.g., trough BFR) might be a promising intervention strategy which is likely to increase the efficiency of resistance training regarding the enhancement of cognitive functions in the following:

(i) On the cellular and molecular level: Some investigations showed a significant higher release of hormones which is associated with positive neurophysiological adaptations, such as serum IGF-1 [144,145], growth hormone (GH) [146,147,148,149] and vascular endothelial growth factor (VEGF) [145,147,150,151], in response to acute resistance activities with BFR when compared to resistance training without BFR. Regarding the IGF-1, also a long-term intervention (two weeks) of low-intensity BFR training which was provided twice a day led to a higher basal level of IGF-1 in comparison to the same resistance training without BFR [152]. As mentioned in the previous section, IGF-1 plays an important role in synaptic functioning and cognitive processes [75]. Because of the link between a deficiency in serum GH level and a cognitive impairment, increases in GH are associated with benefits for cognitive performance [153,154]. Furthermore, in older adults who regularly perform physical exercises, a higher level of GH and better cognitive performance was noticed compared to sedentary older adults [155]. VEGF is involved in angiogenesis [39,156,157,158,159,160,161] and it is speculated that a decrease in angiogenic factors (e.g., serum VEGF) might be associated with cognitive impairments (e.g., in Alzheimer disease) [162,163]. Notably, the increases in neurochemical substances (e.g., IGF-1) was predominantly observed after an acute bout of resistances activities with BFR, thus long-term studies are needed to investigate whether a pronounced release of those neurochemicals would be persistent after longer time intervals (e.g., 6 months).

Furthermore, there is a robust body of evidence suggesting that the blood lactate concentrations are higher after an acute bout of resistance activities with BFR as compared to a resistance exercise without BFR [145,148,149,164,165,166,167,168,169,170]. The levels of post-exercise blood lactate concentration are associated with acute improvements in cognitive functions such as short-term memory [171] and executive functions [172,173]. This phenomenon occurs because peripherally expressed lactate can cross the BBB by monocarboxylate transporters (MCTs) and will be utilized as fuel for cognitive processes due to oxygenation [174,175,176,177,178]. Moreover, lactate is associated with changes in peripheral brain-derived neutrophic factor (BDNF). Here, Ferris et al. [179] showed a correlation between blood lactate concentrations and BDNF [179]. Besides, Schiffer et al. [180] observed an increase in BDNF after a lactate infusion in rest [180]. These insights suggest a potential neurobiological relationship between both neurochemical substances. BDNF is a member of neurotrophins and contributes to neuroplasticity which, in turn, facilitates cognitive performance [181,182].

In addition, systemic hypoxia [183,184] as well as local hypoxia [185] increase the hypoxia-inducible factor 1α (HIF-1α) which is the master regulator for adaptions of oxygen homeostasis. An increase of HIF-1α in response to systemic and/or localized hypoxia (e.g., induced by BFR) might be meaningful for cognition or the integrity of the brain considering the following two aspects: Firstly, the HIF-1α has a neuroprotective effect [186] and secondly, this transcription factor triggers the increase of neurotrophic factors such as the VEGF and IGF-1 [187,188]. Therefore, the HIF-1α may be also a crucial factor for neurocognitive adaptations following a resistance training with BFR.

(ii) On the functional level: After a resistance training with BFR, increases in the cortical excitability [189] and higher levels of oxygenated hemoglobin were observed in cortical motor areas (compared to same resistance exercises without BFR) [190]. Furthermore, in prefrontal areas, a higher concentration of deoxygenated hemoglobin was observed during knee extension with BFR whereas the increase in oxygenated hemoglobin was diminished when compared to knee extensions without BFR [191]. In general, decreased levels of deoxygenated hemoglobin and increased levels of oxygenated hemoglobin are associated with increased cortical activity [192,193,194,195]. Since deoxygenated hemoglobin is assumed to be less affected by physiological artefacts than oxygenated hemoglobin [192,196,197,198,199,200,201], it is perhaps a better proxy of cortical activity (in this particular case) and it could therefore be speculated that a pronounced decrease of deoxygenated hemoglobin may point towards a higher cortical activation during knee extensions with BFR. Nevertheless, further research is necessary to verify or falsify these assumptions.

In general, higher levels of cortical activity (e.g., shown by higher concentration of oxygenated hemoglobin in the brain) after physical exercises are associated with improved cognitive performance [202,203]. It was observed that participants with an improved cognitive performance after exercise showed a higher cortical activity in prefrontal areas during the exercise sessions (termed as responders) in comparison to participants with no cognitive improvements (termed as non-responders) [204]. In consideration of these insights, the enhanced performance in the Stroop test after a low-load resistance training with BFR observed in the investigation of Sardeli et al. [143] may have been caused by higher levels of oxygenated hemoglobin in the prefrontal cortex [143].

### 3.1. Hypothesis

According to the potential neurobiological advantages of a resistance training with BFR compared to a resistance training without BFR on cellular and molecular level as well as on functional level of the brain (see Figure 1C), we hypothesize that a short-term and long-term resistance training with BFR is more efficient regarding the enhancement of cognitive functions than a “traditional” resistance exercise regime without BFR.

### 3.2. Considerations to Evaluate the Hypothesis

To test the hypothesis stated in the previous section, there are a number of general aspects that should be considered regarding (i) the participants’ characteristics, (ii) designing the resistance training program and (iii) the outcome measures.

(i) Regarding the selection of participants, it should be considered that individual characteristics moderate the outcomes and underlying neurobiological processes. Exemplarily, sex is a key moderator for the effect of physical exercise interventions on cognitive performance which is perhaps related to underlying neurobiological processes [116,205,206]. Here, it is assumed that women may benefit more from exercise than men with respect to cognitive functions like executive functions [116]. While the reason for this sex-phenomenon is not fully understood, it is assumed that those findings are related to sex-dependent neurobiological mechanisms (e.g., exercise-induced release of BDNF) and the higher level of habitual physical inactivity in older women (compared to older men) [68,116,205,206]. Another moderator which potentially influences the exercise-cognition interaction is the genotype of the participant [68,116] and through matching the individuals’ genotypes to an appropriate resistance training program, a greater outcome regarding muscular strength can be evoked [207]. However, currently there is not enough evidence available which would allow validly designing resistance training regimes/programs with or without BFR as a function of individual genotypes. Hence, further investigations in this field are needed. Here, moderator and mediator variables should be carefully assessed and their influence on outcomes measures as well as neurobiological processes evaluated. A deeper understanding of moderator and mediator variables would assist the development of more personalized training regimes which may provide greater intervention efficiency [68].

Additionally, further studies should consider and test the “human baseline hypothesis” which proposes that the baseline values of strength (e.g., grip strength and/or knee extensor strength) assessed prior to resistance training or after a detraining period are more appropriate markers of long-term health outcomes compared to training-related strength gains [108]. Therefore, in relation to brain-health gains (brain volume, cognitive functions), the baselines of strength as well as muscle mass should be taken into account.

(ii) For designing resistance training programs, in general, the following exercise variables should be considered [120,208,209]:

Variables of a resistance training session:

(1) *load* (amount of weight that is used for an exercise; usually given as a percentage of the one repetition maximum [1RM]);
(2) number of repetitions;
(3) number of sets;
(4) inter-set rest periods;
(5) inter-exercise rest periods;
(6) *number of exercises* (for the whole training session or for a muscle or a muscle group with the same function);
(7) *repetition velocity* (temporal details should be given for: concentric phase–inter-repetition rest periods–eccentric phase rest period up to the start of the next repetition, e.g., 2–0–2–1 s);
(8) *muscle action* (concentric, eccentric, isometric);
(9) *exercise selection* (e.g., multi-joint or single joint exercises);
(10) *exercise order* (e.g., squat, leg extension, biceps curl and concentration curl or squat, biceps curl, leg extension and concentration curl);
(11) volitional muscle failure
(12) range of motion.

Variables for structuring resistance training:

(13) *frequency* (number of training sessions per week);
(14) *density* (distribution of training sessions across a week with regard to recovery time in-between training sessions) and
(15) *duration* (duration over which a training program is carried out before exercise variables are changed).

It should be noted that some exercise variables are usually summarized into variables with a different designation: e.g., volume (exercise variables 2, 3 and 6) or time under tension (TUT, sum of the exercise variables 2 and 7) [120,209]. Additionally, the cuff pressure is of particular importance in resistance exercises with BFR, as it is intended to induce an appropriate level of localized hypoxia as physiological stimuli [210,211,212,213,214,215,216,217]. Here, the cuff pressure should be applied in such a way that venous pooling without an arterial occlusion would occur. To achieve this, the cuff pressure must be below the arterial occlusion pressure [124]. However, various moderator variables can influence the cuff pressure:

(1) *Cuff width*: wide BFR-cuffs restrict arterial blood flow more than narrow BFR-cuffs using the same cuff pressure. Therefore, the cuff pressure should be applied relative to the cuff width [214,215,218,219,220,221,222].
(2) *Cuff material*: it might be that the cuff material has an impact on the arterial blood flow restriction [211]. However, current investigations comparing different cuff materials (5 cm nylon vs. 3 cm elastic cuffs) do not consider the cuff width [223]. In contrast, Loenneke et al. [224] compared nylon and elastic cuffs with the same width (5 cm) and observed no differences in the arterial occlusion pressure [224].
(3) *Restricted extremity* (upper or lower limbs): cuff pressures should be determined individually for the upper and lower limbs [225].
(4) *Systolic / arterial blood pressure*: the cuff pressure depends on the systolic / arterial blood pressure [213,218,226,227,228,229,230,231,232].
(5) *Body composition / anthropometry*: the circumference of the limbs is the biggest predictor for the cuff pressure to reach arterial blood flow restriction and should be considered [218,225,233,234,235].
(6) *Body position*: the cuff pressure to reach arterial blood flow restriction must be lower in the supine position compared to the seated position and standing position [210,212].
(7) *Exercise protocol*: applying intermittent or continuous pressure; it might be that a BFR applied with a continuous pressure on the cuffs during the exercise leads to another physiological stimulus as compared to a BFR applied in an intermittent fashion [124,226,236,237,238].
(8) *Blood flow restriction system*: different blood flow restriction systems (automatic pressure control vs. manual handheld pressure control) lead to diverging pressure on the limbs at rest and during exercise. However, one first investigation by Hughes et al. [239] compared several blood flow restriction systems with different cuff widths. Therefore, the influence of blood flow restriction systems for inducing effective BFR-stimuli needs further investigations [239].

Since those mentioned moderator variables are crucial for an effective BFR-stimulus and the physiological response, as well as the psychological response, it is likely that those also alter neurocognitive adaptations, which, in turn, influence the changes in cognitive functions. To evoke the above mentioned cognition-related neurobiological adaptations through a resistance training with BFR, it is strongly recommended to determine a personalized cuff pressure be chosen [217,240] which takes the above mentioned relationships of the moderator variables and the cuff pressure into account. From a practitioner’s view, the optimal solution(s) to determine the cuff pressure ensuring an appropriate stimulus would be using a pressure that is relative to the used cuffs and individual’s characteristics [117,241] or to use a BFR system that automatically adjust the cuff pressure [239,240]. Furthermore, even moderate cuff pressures induce adaptions comparable to high cuff pressures [227,242]. Hence, moderate cuff pressures are recommended because higher cuff pressures increase the risk of full arterial occlusion and in turn of adverse effects [131,243,244].

In resistance training without BFR, only the following exercise variables are considered and recommended to enhance cognitive functions, by now (to 1.) load: 60 to 80% of 1RM; (to 2.) number of repetitions: 7; (to 3.) number of sets: 2; (to 4.) inter-set rest periods: 2 min; (to 13.) frequency: at least twice per week; (to 15.) duration of a training period 2 to 12 months [61]. However, in short-term and long-term resistance training interventions with (and even without) BFR, the optimal selection of exercise variables to efficiently enhance cognitive functions are largely unknown, and should be investigated in future studies. Nevertheless, we would like to recommend the following exercise variables for a resistance training with BFR aiming to induce neurocognitive adaptations (Table 1). We chose these exercise variables because of their effectiveness to increase muscular strength as well as muscular hypotrophy [124,130,137,144,216,245,246,247,248]. As described above, functional and structural adaptations of the musculature are moderating factors for the neurocognitive status. Furthermore, based on our above mentioned deliberations, it can be assumed that these exercise variables are efficient to trigger adaptations on the above mentioned neurocognitive levels (cellular, molecular, structural and functional level).

Furthermore, so far, there have been no consistent recommendations for the cuff pressure. However, the following criteria are often used to apply an optimal cuff pressure: 130% of the systolic blood pressure [226,237]; 10 mm Hg below the arterial occlusion pressure [225]; ~50% arterial occlusion pressure [243]. The most effective cuff pressure has still to be identified [124].

In general, resistance training with BFR is a harmless treatment strategy when applied appropriately [117,133,136,222,241,249,250,251], but in order to minimize the occurrence and/or to avoid adverse health effects, safety recommendations should be considered [134] and available risk assessment tools should be used [252]. Furthermore, we want to point out that during the practical implementation of a resistance training with BFR, the following general safety recommendations should be strictly adhered to minimize the occurrence of adverse events: We strongly recommend that an individual and adequate cuff pressure should be applied. Furthermore, based on the currently available recommendations the maximal duration for continuous BFR should in general not exceed a time period of circa 10 to 15 min for the upper limbs and circa 15 to 20 min for the lower limbs because longer time periods may increase the risk of adverse events [134].

(iii) Physical exercises influence cognitive performance on multiple levels: (1) cellular and molecular level; (2) structural and functional level and (3) behavioral/socioemotional level [67]. Based on these mentioned levels, multiple outcome measures should be considered in the study design and analysis in order to understand the complex interaction of physical exercises (e.g., resistance training with BFR) and cognition:

(1) On the cellular and molecular level, neurochemical markers such as IGF-1, GH, VEGF, blood lactate concentrations and BDNF might be used since the exhibited associations with cognitive performance (see the previous sections).

(2) On the structural and functional level, different neuroimaging modalities such as fNIRS, EEG, fMRI or a combination of those should be applied in order to understand physical exercise induced structural and functional brain changes [49,253]. Since fNIRS and EEG can in particular be used during physical exercises [254,255,256], both measuring systems are suitable for the evaluation of cognitive activity while performing resistance training with BFR. Here, short-term and long-term effects of this intervention strategy could be objectified. Regarding functional brain changes, it seems recommendable (a) to use standardized and established cognitive test (e.g., Stroop test [101,104], Sternberg test [257,258,259], Eriksen Flanker test [102]) to ensure comparability with existing studies and (b) to consider attention and perceptual tasks which were currently not in the focus of exercise-cognition research [260] but could be important for special cohorts (e.g., individuals with dementia) [48].

(3) On the behavioral/socioemotional level, established questionnaires such as “Felt Arousal Scale” [261], “Ratings of perceived exertion” [262], “Visual Analogue Scales” (e.g., to assess motivation or mental fatigue) [172,263,264,265], “SF-36” (to assess physical and mental components of the quality of life) [266] and “Pittsburgh Sleep Quality Index” (to assess various components of the sleep quality) [267] which are widely used in exercise–research with a neuropsychological and behavioral/socioemotional focus [141,172,263,264,265,268,269], should be used to elucidate the (moderating) effects of socioemotional states.

## 4. Conclusions

The type of physical exercise (e.g., resistance training) in combination with related exercise variables (e.g., load, number of repetitions and sets) which efficiently enhance cognitive performance are largely unknown [39,40,41,42,43,44,45,46,47,48,49]. A promising physical exercise intervention which fends off physical and cognitive decline (e.g., due to the aging process) is resistance training. Hypothetically, the efficiency of resistance training interventions on cognition could be increased due to the application of BFR.

Resistance training with BFR is more efficient to increase muscle hypertrophy and strength as compared to the same resistance training without BFR [247,270] and for a resistance training with BFR, lower exercise loads are needed to achieve comparable muscular adaptions (e.g., increase in muscle mass) as compared to high-load resistance training [271,272]. The lower exercise load during a resistance training with BFR could be beneficial for special cohorts since those lower exercise loads pose lower mechanical stress to the joints and the risk of adverse cardiovascular effects is decreased [124,217,244,273]. The currently available evidence suggests (i) that strength gains in response to a resistance training mediate, at least partly, the cognitive improvements [274] or (ii) that strength performance per se is a more appropriate indicator regarding health outcomes (e.g., cognition) [108]. Hence, at the moment no reliable assumptions can be made whether (i) a regular participation in resistance training, (ii) a relative high individual (baseline) strength level or (iii) the combination of both (high muscular strength level and regular resistance training) are most beneficial for cognitive functions. Notably, since an optimal level of neurochemical substances (e.g., IGF-1) is beneficial for cognitive performance [275], it could be speculated that, in turn, also an optimal level of muscular strength and/or continuously performed effective resistance activities, which may contribute substantially to the maintenance of an optimal level of neurochemical substances, exists. In this manner, a low-load resistance training with BFR could be a promising strategy especially for special cohorts (e.g., older adults unable to tolerate high loads) to ensure an adequate level of strength and profit from biological mechanisms which would without BFR only be possible when (not well tolerated) high loads are applied. Furthermore, relative low muscle damage is induced by low-load resistance training with BFR [148,168,276,277], which may allow a higher training frequency than in high load-resistance training [124,125,246].

However, testing the hypothesis suggesting that short-term and long-term resistance training with BFR improve cognitive performance as well as brain health to a greater extent than resistance training without BFR may provide deeper insights into the interplay between neurobiological mechanisms and cognitive processes. A deeper understanding of underlying exercise-induced and cognition-related neurobiological mechanisms is urgently needed to develop efficient prevention strategies (e.g., decelerate cognitive decline due to aging process) and to optimize rehabilitation strategies for individuals with worsened cognitive functions (e.g., older individuals with dementia). Here, the resistance training with BFR might be a promising strategy of exercise intervention.

## Figures and Tables

**Table 1:** Recommendations for exercises variables for a resistance training with blood flow restriction (BFR); n.a.: not available; reps: repetitions; 1RM: one repetition maximum; s: seconds; min: minute.

Exercise Variables	Recommendations for Resistance Training with BFR
(1.) load	20 to 50% of 1RM
(2.) number of repetitions	15 to 30 per set, 50 to 80 repetitions per exercise (e.g., 30–15–15–15 reps)
(3.) number of sets	3 to 5 sets per exercise
(4.) inter-set rest periods	30 to 60 s
(5.) inter-exercise rest periods	5 min (without BFR)
(6.) number of exercises	n.a.
(7.) repetition velocity	1 (to 2)–0–1 (to 2)–1 s
(8.) muscle action	dynamic muscle action, eccentric is more effective than concentric
(9.) exercise selection	single- and multi-joint exercise
(10.) exercise order	n.a., depending on the training goal
(11.) volitional muscle failure	until volitional fatigue/repetition failure/technical failure
(12.) range of motion	full range of motion
(13.) frequency	2 to 3 sessions per week
(14.) density	n.a., depending on the performance level
(15.) duration	n.a., but according to the general physiological view, exercise variables or exercises should be changed after a mesocycle of 8 to 12 weeks
